# Characterization and expression profile of *CaNAC2* pepper gene

**DOI:** 10.3389/fpls.2015.00755

**Published:** 2015-09-17

**Authors:** Wei-Li Guo, Shu-Bin Wang, Ru-Gang Chen, Bi-Hua Chen, Xiao-Hua Du, Yan-Xu Yin, Zhen-Hui Gong, Yu-Yuan Zhang

**Affiliations:** ^1^College of Horticulture, Northwest A&F UniversityYangling, China; ^2^School of Horticulture Landscape Architecture, Henan Institute of Science and TechnologyXinxiang, China; ^3^Institute of Vegetable Crops, Jiangsu Academy of Agricultural SciencesNanjing, China

**Keywords:** *Capsicum annuum* L., *CaNAC2*, expression profile, abiotic stress, virus-induced gene silencing

## Abstract

The plant-specific NAC (NAM, ATAF, and CUC) transcription factors have diverse role in development and stress regulation. A new transcript encoding NAC protein, homologous to nam-like protein 4 from *Petunia* was identified from an ABA-regulated subtractive cDNA library of *Capsicum annuum* seedling. Here, this homolog (named *CaNAC2*) from *C. annuum* was characterized and investigated its role in abiotic stress tolerance. Our results indicated that a plant-specific and conserved NAC domain was located in the N-terminus domain of *CaNAC2* which was predicted to encode a polypeptide of 410 amino acids. Phylogenetic analysis showed that *CaNAC2* belonged to the NAC2 subgroup of the orthologous group 4d. The protein CaNAC2 was subcellularly localized in the nucleus and it had transcriptional activity in yeast cell. *CaNAC2* was expressed mainly in seed and root. The transcription expression of *CaNAC2* was strongly induced by cold, salt and ABA treatment and inhibited by osmotic stress and SA treatment. Silence of *CaNAC2* in virus-induced gene silenced pepper seedlings resulted in the increased susceptibility to cold stress and delayed the salt-induced leaf chlorophyll degradation. These results indicated that this novel *CaNAC2* gene might be involved in pepper response to abiotic stress tolerance.

## Introduction

Pepper (*Capsicum annuum* L.), the *Solanaceae* family, is an important spicy crop. Pepper plants originate from tropical regions and the optimal temperature for its growth ranges between 21 and 27°C (Deng et al., 2009). Low temperature severely affects the growth and reproduction of pepper plants, resulting in economic losses (Pressman et al., 2006; Korkmaz et al., 2010; Schwarz et al., 2010). Cold stress has created a great threat to the vulnerability of pepper. To respond to low temperature stress, plants have caused many physiological, and biochemical modifications to adapt to stress condition (Thomashow, 1999; Shinozaki et al., 2003). Such changes are intricately regulated by genes t. Among these regulating genes, plant transcription factors play an important role in the activation of genes related with stress tolerance.

Plant-specific NAC transcription factors comprise a large gene family. A highly conserved N-terminal of these family proteins contains five sub-domains (A–E) and has the DNA binding ability of NAC proteins. The C-terminal section of NAC proteinsis diverged and potentially activates transcription (Ernst et al., 2004). A number of studies have revealed plant NAC proteins to be involved in transcriptional regulation in various processes. Some are related to the development of the shoot apical meristem, lateral root formation, senescenceand secondary wall formation (Xie et al., 2000; Duval et al., 2002; Olsen et al., 2005). Other NAC proteins have been implicated in response to various abiotic stresses, including abscisic acid (ABA), ethylene, drought, high salt, and low temperature (Tran et al., 2004; He et al., 2005; Hu et al., 2008). In *Arabidopsis*, the expression of *ANAC019, ANAC055*, or *ANAC072* is induced by ABA, drought, salinity or cold stress and over expression of these genes in transgenic plants confers a constitutive increase in stress tolerance (Fujita et al., 2004; Tran et al., 2004). *Arabidopsis AtNAC2* is also induced by salinity, ABA, and it has been predicted to be involved in the ethylene and auxin signaling pathways as a down-stream gene (He et al., 2005). In rice, some *NAC* genes have been characterized to be involved in responses to environmental stimuli (Hu et al., 2006, 2008; Jeong et al., 2010). More recently, in the *Solanaceae* family, *Solanum tuberosum StNAC* gene isup-regulated by *Phytophtora* infestans infection (Collinge and Boller, 2001), *C. annuum CaNAC1* gene is induced by bacterial pathogen and SA treatment (Oh et al., 2005), and *SlNAC1* and *SlNAM1* genes from *S. lycopersicum* are up-regulated by salinity (Yang et al., 2011) and another *SlNAC3* is down-regulated by drought, salinity and ABA (Han et al., 2012). Therefore, pepper NAC transcription factors associated with stress tolerance particularly need further to be investigated.

In order to divulge the mechanism involved in cold-tolerance, suppression subtractive hybridization (SSH) revealed that ABA-mediated candidate genes have been fully identified in pepper seedlings subjected to cold stress (Guo et al., 2013). One of the genes cloned from the forward subtraction was homologous to *Petunia* nam-like protein 4 encoding NAC transcription factor. Expression of this NAC homolog was significantly induced by cold stress and ABA. However, the involvement of this gene in the stress tolerance remains to be investigated. Here, we reported the characterization of a novel pepper NAC transcription factor gene designated as *CaNAC2*. Expression profiles in response to abiotic stress, and ABA, SA treatment were also examined by quantitative real time-PCR (qRT-PCR). Furthermore, the function of this gene silencing in pepper plants was studied by a virus-induced gene silencing (VIGS) method. These findings suggest that *CaNAC2* in pepper could be involved in defense response against abiotic stress.

## Results

### Cloning and Sequence Analysis of *CaNAC2* Gene

Pepper cold-related candidate genes from SSH library was reported previously (Guo et al., 2013). One of the isolated clones showed 82% homologous to nam-like protein 4 from *Petunia*. This homolog was used as the initial probe to screen the *Solanaceae* EST database in GenBank and the full-length clone was isolated by a homology-based candidate gene method. We designated it *CaNAC2* followed with *CaNAC1* that had been functionally identified in *C. annuum* (Oh et al., 2005). The entire cDNA fragment was 1,490 bp in length and contained a single open reading frame (ORF) of 1,230 bp (GenBank accession No. JX402928, **Supplementary Figure S1**). It encoded 409 amino acids with 45.80 kDa molecular mass and a theoretical isoelectric point of 5.34.

**FIGURE 1:**
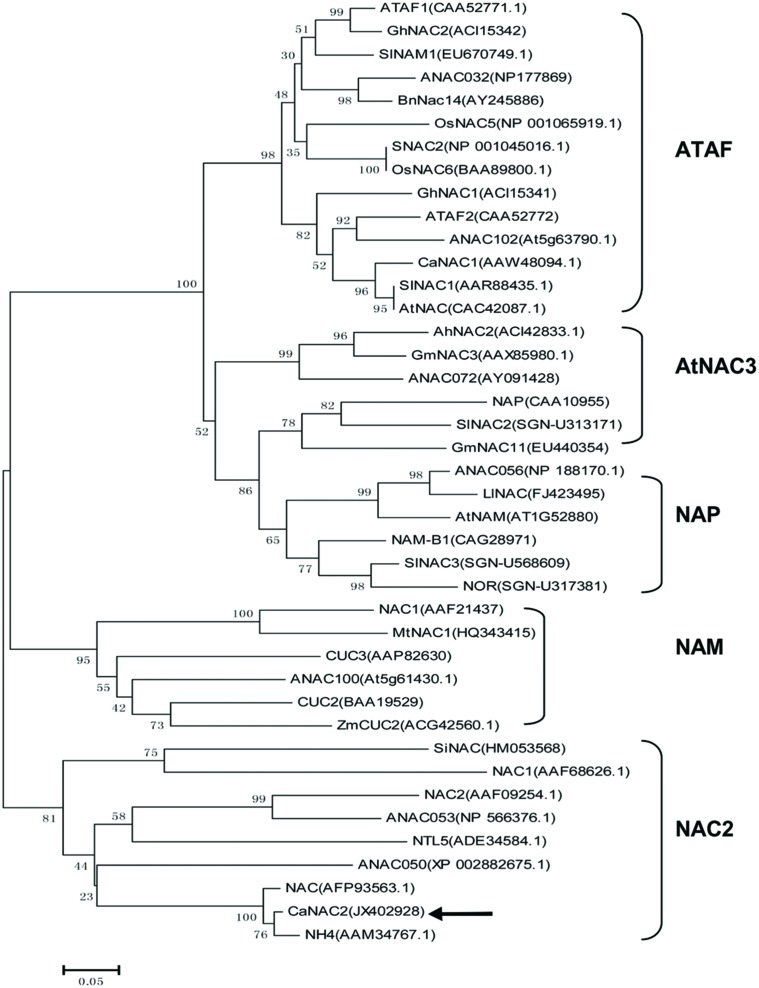
**A Neighbor-joining phylogenetic tree of CaNAC2 and NAC proteins from different plant species.** The GenBank accession No. of NAC proteins are shown in the brackets. The CaNAC2 protein is marked with arrow. The NAC subgroup names are shown to the right of square.

A phylogenetic tree was conducted between the overall amino acid sequences of CaNAC2 and other known NAC-domain proteins. CaNAC2 was clustered into the NAC2 subgroup (**Figure 1**) and highly homologous to NH4 protein from *Petunia* (84%, identity). Whereas, CaNAC2 was low homologous to ANAC053, ANAC050, and NAC2 from *Arabidopsis*, with similarity of 30, 42, and 26%, respectively (data not shown).

To further analyze the NAC domain of CaNAC2, the overall amino acid sequences of NAC protein were analyzed for alignment. Result showed that the N-terminal region of CaNAC2 contained a conserved NAC domain (29–200 aa) and was divided into five sub-domains (A–E), while the C-terminal was a diversified transcription regulatory region (TRR; **Supplementary Figure S2**). A putative nuclear localization signal (NLS) was predicted at the N-terminus (132–158 aa). The C-terminal regions contained several putative a-helical motifs that did not form transmembrane motifs, structurally distinct from NTLs (NAC with transmembrane motif 1-like; Kim et al., 2007).

### Subcellular Localization and Transcription Activation Assay of CaNAC2 Protein

**FIGURE 2:**
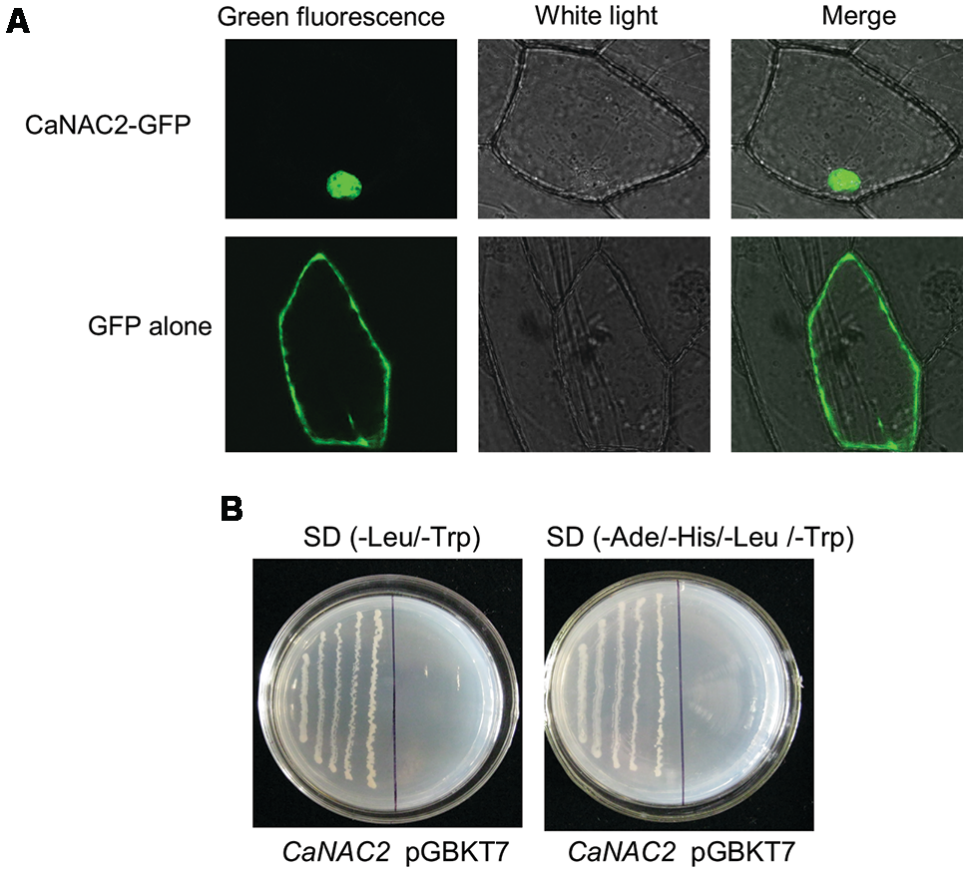
**The subcellular localization and transcriptional activation analysis of pepper *CaNAC2*. (A)** The subcellular localization of pepper *CaNAC2* in onion epidermal cells. The fused pBI221-GFP-*CaNAC2* and pBI221-GFP constructs were introduced into onion epidermal cells by biolistic bombardment. The GFP signals were observed under confocal microscope; **(B)** Transcriptional activation analysis of *CaNAC2* in yeast strain AH109. *CaNAC2* represents the fusion protein of the GAL4 DNA-binding domain and CaNAC2; pGBKT7 was used as control. The culture solution of the transformed yeast was streaked on SD/-Leu/-Trp medium and SD/-Ade/-His/-Leu/-Trp medium. The plates were incubated for 3 days.

CaNAC2 was predicted to be nucleus localized by ProComp v 8.0 program. To confirm this prediction, the subcellular localization of CaNAC2-GFP fusion protein was demonstrated by fusing the entire CaNAC2 coding sequence to GFP and expressed them under the control of the CaMV35S promoter in the onion epidermal cells. Cells expressing GFP alone displayed diffuse cytoplasmic and nuclear staining (**Figure 2A**) supported by Guo et al. (2015), while the GFP-CaNAC2 full length protein was located only in the nucleus and the 132–158 amino acids containing the putative NLS can mediate the nuclear targeting of the protein.

To validate that CaNAC2 functions as a transcriptional activator, the ORF of CaNAC2 was fused to the GAL4 DNA binding domain (GAL4-DB) in the vector pGBKT7 and the construct was transformed into yeast strain AH109 (**Figure 2B**). Colonies transformed with pBD-CaNAC2 construct grew on both SD/-Leu/-Trp and SD/-Ade/-His/-Leu/-Trp unlike the vector-transformed colonies, indicating that the CaNAC2 had transcriptional activity in yeast cell.

### Expression Patterns of *CaNAC2* Under Abiotic Stresses

**FIGURE 3:**
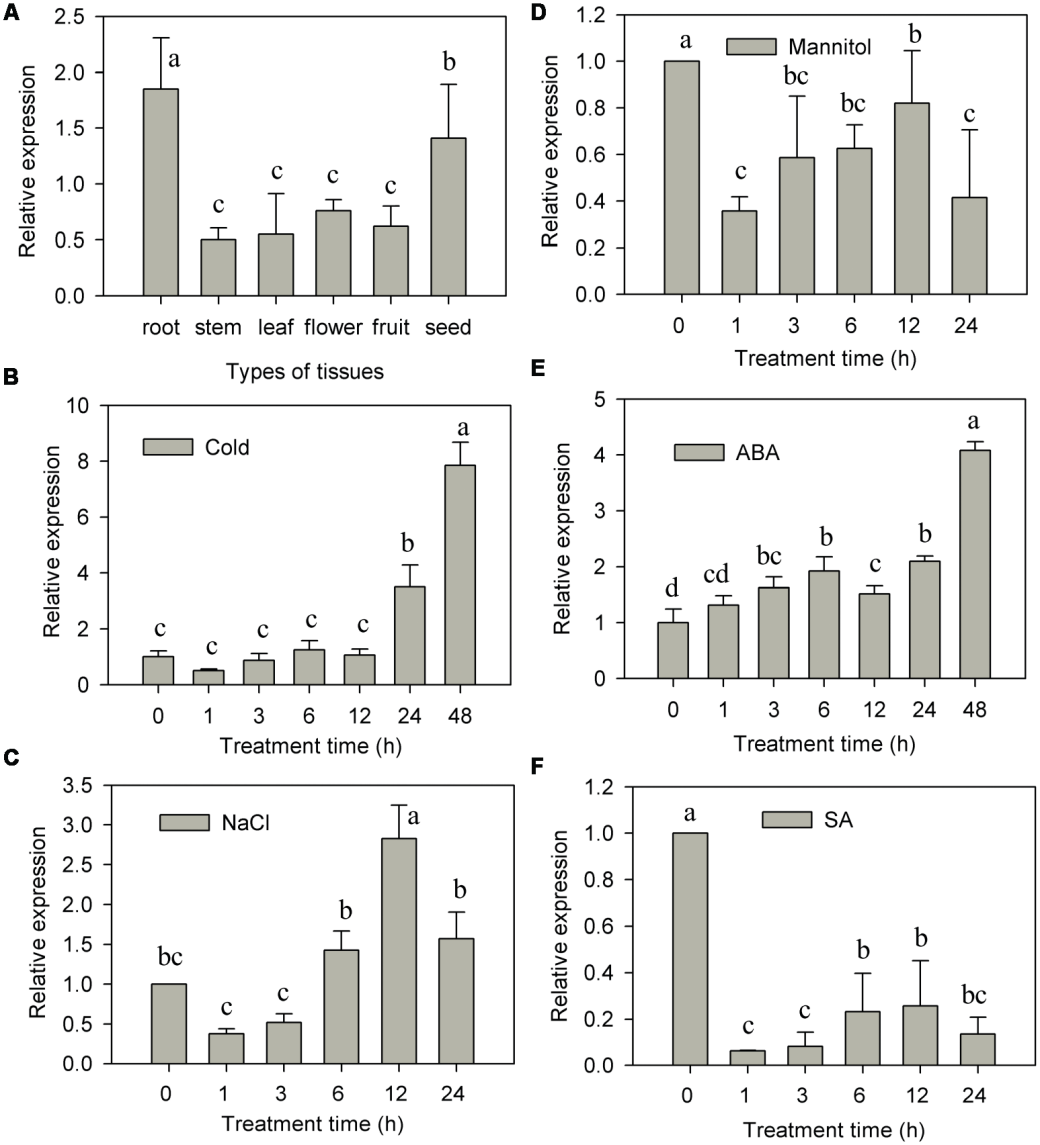
***CaNAC2* expression both in tissue and in response to stress and exogenous hormones. (A)** Tissue specific expression of *CaNAC2*; **(B)** The pepper seedlings were exposed to cold stress (6°C), **(C)** salt stress (300 mM NaCl) and **(D)** osmotic stress (300 mM mannitol) for the indicated times; the pepper seedlings were sprayed with exogenous ABA **(E)** and SA **(F)**. Pepper Ca*Ubi3* gene was used as an internal control for qRT-PCR. The transcript level of *CaNAC2* at 0 h is used as control (quantities of calibrator) and was assumed as 1. Results are the mean ± SE. Different letters indicate that the mean values are different by the Tukey HSD test (*p* ≤ 0.05). Each mean was compared with all the other mean values shown in the same figure.

The expression pattern of *CaNAC2* in different pepper tissues were detected by qRT-PCR. This analysis revealed that the transcription of *CaNAC2* was abundant in root and seed, low in stem, leaf, flower, and fruit (**Figure 3A**).

The transcription expression of *CaNAC2* was further investigated by abiotic stresses and ABA, SA treatments in seedlings. When exposed to cold stress, the *CaNAC2* transcript had no obvious changes within 12 h, significantly increased at 24 h and peaked up to eightfold at 48 h (**Figure 3B**). The transcript of *CaNAC2* initiated to increase within 6 h, and reached the maximum after 12 h of salt stress (**Figure 3C**). Steady-state transcript of *CaNAC2* reached to maximum after 48 h of ABA alone treatment (**Figure 3E**). Meanwhile, the expression of *CaNAC2* was distinctly repressed by mannital and SA treatments (**Figures 3D,F**).

### *CaNAC2*-Silenced Pepper in Response to Chilling Stress and Salt Stress

**FIGURE 4:**
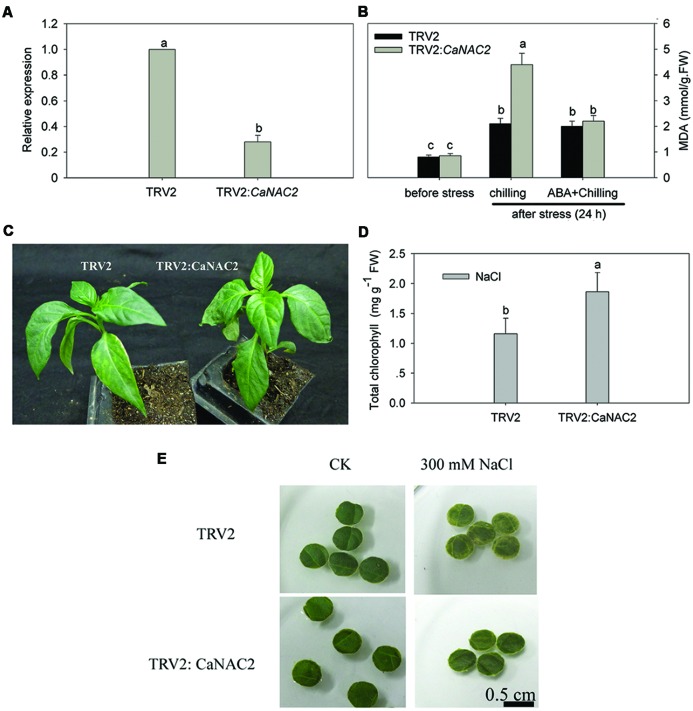
***CaNAC2*-silenced pepper plants. (A)** The expression of *CaNAC2* in gene-silenced pepper (TRV2: *CaNAC2*) cv P70 and control plants (TRV2:00) were tested at 45 days after inoculation; **(B)** Effect of ABA application and chilling stress on lipid peroxidation (MDA) content in *CaNAC2*-silenced pepper seedlings. **(C)** Phenotypes analysis of the *CaNAC2*-silenced and non-silenced pepper seedlings under 6°C cold stress for 6 h. **(D)** Chlorophyll contents of leaf disks. **(E)** Leaf disks phenotypes. Leaf disks from the gene-silenced plants were floated in 300 mM NaCl solutions for 72 h at 25°C. Bars with different lower case letters indicate significant differences compared to empty vector control leaves (Student’s *t*-test, *p* ≤ 0.05).

Compared to the negative control (inoculated with TRV2), the *CaNAC2* silencing rate reached nearly 72% (**Figure 4A**), suggesting that VIGS was successful and effective for *CaNAC2* gene silencing in pepper. About 35 silenced pepper seedlings were used for chilling stress, ABA pre-treatment followed by chilling stress or salt stress. Loss-of-function of *CaNAC2* in pepper plants increased susceptibility to chilling stress, the lipid peroxidation (MDA) content of the *CaNAC2*-silenced leaves was significantly higher than that of the empty vector control plants. Chilling stress after pretreated with ABA, there is no significant difference between the negative control and the silenced pepper in the MDA content (**Figure 4B**). Obvious wilting appeared after 6 h of chilling stress in *CaNAC2*-silenced plants, while control leaves did not exhibit withering (**Figure 4C**). Responding to 300 mM salt stress after 72 h, the chlorophyll content of the *CaNAC2*-silenced leaf disks was significantly higher than that of the negative control plants (**Figure 4D**) and the leaf disks of *CaNAC2*-silenced pepper stayed green while the negative control appeared loss of chlorophyll (**Figure 4E**).

## Discussion

In this study, a novel pepper *NAC* gene with full-length cDNA sequence, designated as *CaNAC2*, was isolated. The phylogenetic tree was constructed that CaNAC2 belonged to NAC2 subgroup (**Figure 1**) and further divided into the orthologous group 4d (Ooka et al., 2003; Cenci et al., 2014). The C-terminal regions of other members from this group contained transmembrane motifs, such as SINAC, ANAC053, and TaNTL5 (Puranik et al., 2011; Lee et al., 2012; Tang et al., 2012). The TMpred program predicted that the C-terminal of CaNAC2 did not form transmembrane motifs, structurally distinct from NTLs (details not shown). This phylogenetic tree analysis indicated the extremely functional diversification of *NAC* gene subfamily in plant.

Sequence analysis predicted NLS at the N-terminus of CaNAC2 (**Supplementary Figure S2**). The subcellular localization assay demonstrated that the CaNAC2 was targeted to the nuclei (**Figure 2A**), the nuclei localization of which was different from NTLs of NAC2 subfamily. These results also proved that the C-terminal of CaNAC2 did not contain transmembrane motifs. Previous reports were shown that many NTLs proteins localized in cytoplasm and nucleus (Puranik et al., 2011; Lee et al., 2012; Tang et al., 2012). Results of transcription activation assays in yeast cells demonstrated that CaNAC2 encoded a NAC transcription activator, which had transcription activity (**Figure 2B**). The TRR of NAC TFs, generally lying at the highly diverged C-terminal, can activate transcription.

Tissue-specific transcription factor plays an important role in plant development and growth (He et al., 2005). In the present study, *CaNAC2* was expressed in all tested tissues and high level in root and seed (**Figure 3A**), suggesting that CaNAC2 can play an important role in root growth and seed maturation. *AtNAC2* was mainly expressed in root and can promote lateral root development (Xie et al., 2000). *CarNAC1* was induced during seed development and germination (Peng et al., 2010). Many NAC family members have shown different responses to abiotic stress. For example, rice *OsNAC6* was induced by low temperature, high salt, drought, and ABA treatment (Ohnishi et al., 2005; Nakashima et al., 2007), and over expression of this gene in rice can improve the tolerance of stress (Nakashima et al., 2007). *BnNACs* from *Brassica campestris* was also reported to be increased by multiple stresses such as wound, cold, and drought stress (Hegedus et al., 2003). Soybean nine *NAC* genes were induced by one or several treatments of drought, high salt, low temperature, or ABA (Tran et al., 2009). Furthermore, over expression of one of these genes (*GmNAC20*) enhanced salt and freezing tolerance in transgenic *Arabidopsis* plants (Hao et al., 2011). It has been reported that NAC transcription factors respond in both ABA-dependent and -independent pathways to abiotic stress (Fujita et al., 2004; Tran et al., 2004). In this study, *CaNAC2* was significantly induced by cold stress, salt, and ABA treatment (**Figures 3B–E**), indicating that this gene was involved in the response to cold and salt stress by ABA signaling. Meanwhile, *CaNAC2* described here was strongly suppressed by osmotic stress (**Figure 3D**) and SA treatment (**Figure 3F**). Lipid peroxidation were more severely influenced by chilling stress in *CaNAC2*-silenced leaves compared with empty vector control plants (**Figure 4B**), indicating that silence of *CaNAC2* in pepper plants increase sensitivity to cold stress. That result was consistent with the visible symptoms of leaf damage in *CaNAC2*-silenced seedlings subjected to chilling stress (**Figure 4C**). In contrast, the leaf disks of *CaNAC2*-silenced pepper increased the chlorophyll content and appeared green under salt stress (**Figures 4D,E**). These results suggested that *CaNAC2* is involved in the salt-induced chlorophyll degradation, which are different from that of the increased susceptibility to cold stress. The difference might be related to the imposed stress condition. Since different stresses may damage plant growth and development in specific ways, the plant could alleviate damage by different mechanisms.

In conclusions, *CaNAC2* was highly expressed in seed and root. The expression of *CaNAC2* was significantly induced by cold stress, salt, and ABA treatment and suppressed by osmotic stress and SA treatment. These findings suggested that *CaNAC2* may be involved in response to various abiotic stresses. Silence of *CaNAC2* led to increase susceptibility to cold stress and delayed the salt-induced leaf chlorophyll degradation, suggesting its possible role in stress tolerance. Currently, the authors are further studying the biological functions of *CaNAC2* in response to abiotic stress using over expression method in pepper.

## Experimental Section

### Plant Materials and Stress Treatments

Pepper (*C. annuum* L.) cv P70 seeds were grown using a previously described method (Guo et al., 2012). The six-leaf seedlings were used to establish the following treatments. ABA and cold treatments were performed as described by Guo et al. (2013). For ABA and cold treatments, seedlings were sprayed with freshly prepared 0.57 mM ABA solution or water (control). At 72 h after foliar application, control and ABA treatment groups were subjected to cold stress at 6°C. For salt and osmotic treatments, the seedling roots were immersed in solutions containing 300 mM sodium chloride (NaCl), or 300 mM mannitol. For SA treatment, seedlings were sprayed with 5 mM SA solution and maintained at 25°C for the indicated times. The treated seedlings were harvested after 0, 1, 3, 6, 12, 24, and 48 h for examination of *CaNAC2* expression pattern under various stress conditions. At each time point, two or three upper young leaves from four separate seedlings were collected to form one sample, wrapped with foil, immediately frozen in liquid nitrogen and stored at -80°C. The treatments were arranged in a randomized complete block design (RCBD) with three biological replicates.

### Isolation of *CaNAC2* cDNA Clone and Sequence Analysis

The NAC-homologous EST (GenBank accession No. JZ198750) isolated from a cold-related pepper seedling SSH library was reported by Guo et al. (2013). The full-length ORF of the NAC homolog was obtained using the cDNA fragment of this homolog as a probe by a homology-based candidate gene method (Guo et al., 2014). The full-length forward and reverse primers for *CaNAC2* were 5′-TTCTTCCTTTTTATTTATCTGTG-3′ and 5′-GGTACAGGTTATAGCGAAA -CTACG-3′, respectively.

The theoretical molecular weight (Mw) and isoelectric point (pI) were calculated with the ExPASy compute pI/Mw tool (Bjellqvist et al., 1993). Sequence data were analyzed using Clustal W (Thompson et al., 1994). The phylogenetic tree was constructed using Mega5.0 by the neighbor-joining method. Homology searches in database were carried out using the default parameters of the BLAST program on the website http://www.ncbi.nlm.nih.gov/blast (Altschul et al., 1997).

### Real-Time Quantitative PCR (qRT-PCR) Analysis

Gene-specific primers were designed using ProbeFinder Version 2.44^1^. Primer specificity was then confirmed by blasting each primer sequence against Phytozome^2^ using BLASTN algorithm. RNA extraction, cDNA preparation, and qRT-PCR were performed as described by Guo et al. (2013). Total RNA was extracted using a TRIZOL reagent (Invitrogen, USA). DNA contamination was removed from the RNA samples using DNaseI RNase-free (50 U/μl, Fermentas). The quality and purity of the preparation were determined at OD_260_:OD_280_ nm absorption ratio (1.8–2.0) and the integrity of the preparations was ascertained by electrophoresis in a 1.2% agarose gel containing formaldehyde. And the first-strand cDNA synthesis was performed using the DNase-treated total RNA with a PrimeScript^TM^ first-strand complementary DNA (cDNA) Synthesis Kit (TaKaRa, Japan). qRT-PCR was performed with an iCycler iQ^TM^ Multicolor PCR Detection System (Bio-Rad, Hercules, CA, USA). Relative gene expression levels were determined using the 2^-ΔΔCT^ method. Total RNA was extracted from the leaves of pepper plants subjected to various stress for 0, 1, 3, 6, 12, 24, and 48 h as described above. The ubiquitin-conjugating protein gene (*CaUbi3*, GenBank accession No. AY486137.1) from pepper plants was amplified as a reference gene for normalization of *CaNAC2* cDNA samples (Wan et al., 2011). The corresponding specific primers were listed in **Table 1**.

**Table 1:** Primers used in this investigation.

Gene	Forward (F) and Reverse (R) primer 5′→3′
RT*-CaNAC2*	F: CCGACCTCTGACGTTTGTTTG
	R: AGTTTCCTCAAGTCCTCGTTC
GFP-CaNAC2	F: GGAAATGGAGCAAGAAGGA
	R: CGGGGTACCACCATTTCGGACGAGTTTC (*Kpn*I digest site)
pGBKT7-*CaNAC2*	F: TTCTTCCTTTTTATTTATCTGTG
	R: GGTACAGGTTTATAGCGAAACTACG
*UBI-3*	F: TGTCCATCTGCTCTCTGTTG
	R: CACCCCAAGCACAATAAGAC

### Subcellular Localization Analysis of *CaNAC2*

The ORFs of CaNAC2 cDNAs (without termination codon) were ligated into the pBI221-GFP vector, resulting in an in-frame fusion protein of *GFP* gene and the CaNAC2 ORFs. The construct (pBI221-CaNAC2) and the control vector (pBI221 -GFP) were transformed into onion epidermal cells by particle bombardment using a Biolistic PDS-1000/He gene gun system (Bio-Rad, Hercules, CA, USA). After 24 h at 25°C incubation of transformed onion epidermal cells, GFP signal was detected by a confocal fluorescence microscope (LSM510 Meta; Zeiss, Jena, Germany). The primers flanked with restriction sites for subcloning are listed in **Table 1**.

### Transcriptional Activation Analysis in Yeast

To investigate the transcriptional activation, the CaNAC2 ORFs were fused in-frame with GAL4 DNA-binding domain in pGBKT7 to construct vectors. The pGBKT7 was used as a negative control. These different constructs were transformed into yeast strain AH109. The transformants were streaked on the selective medium lacking Leu and Trp (SD-Leu/-Trp) and selective medium lacking Trp, His, Leu, and Ade (SD-Ade/-His/-Leu /-Trp) medium. After incubation at 28°C for 3–5 days, the growth status of the transformants was evaluated. Primers for PCR amplification are provided in **Table 1**.

### VIGS Assay of *CaNAC2* in Pepper Plants

The TRV-based VIGS system was used for gene silencing, as described previously (Wang et al., 2013). To generate the *CaNAC2*/TRV2 construct, a 447-bp fragment of the *CaNAC2* gene was PCR amplified from pepper. The resulting product was cloned into the TRV2 vector using the double digested method with enzymes of *XbaI* and *BamHI*. Forward primer (5′-GCTCTAGACCGACCTCTGACGTTTGTTTG-3′) with *XbaI enzyme site* and reverse primer (5′-CG CGGATCCAGTTTCCTCAAGTCCTCG TTC-3′) with *BamHI* enzyme site were used for vector construct. *Agrobacterium tumefaciens* strain GV3101 harboring pTRV1 was, respectively, mixed with pTRV2 (as the negative control), TRV2-*CaPDS* (as the positive control) or TRV2-*CaNAC2* at a 1:1 ratio. The mixtures were inoculated into pepper cv P70 at the fully expanded cotyledons stage. After injection, all of the seedlings were placed at 18°C and 60% relative humidity for 2 days and then moved to a growth chamber according to the protocol. Leaf disks were excised from the gene-silenced pepper leaves using a cork borer (0.5 cm in diameter) and were floated in 300 mM NaCl solution at 25°C to test for salt stress. Total chlorophyll contents were spectrophotometrically measured after extracting into 80% (v/v) acetone (Guo et al., 2013). The level of lipid peroxidation was measured in terms of malondialdehyde (MDA), followed the procedure of Guo et al. (2012).

### Statistical Analyses

**V**alues were expressed as the mean ± SE. The data were analyzed using analysis of variance (ANOVA), and the mean separation was analyzed using the Tukey Honestly Significant Differences (HSD) or Student’s *t*-test (*p* ≤ 0.05) using the SPSS Base 12.0 for Windows (SPSS Inc., USA). A *p*-value ≤ 0.05 was considered significant.

## Author Contributions

W-LG. and R-GC conceived and designed the experiments; W-LG and B-HC performed the experiments; X-HD and Y-XY analyzed the data; Z-HG and S-BW contributed reagents/materials/analysis tools; W-LG and Y-YZ wrote the paper.

## Conflict of Interest Statement

The authors declare that the research was conducted in the absence of any commercial or financial relationships that could be construed as a potential conflict of interest.

## Footnotes

1 http://www.roche-applied-science.com.

2 http://www.phytozome.net/search.php?show$=$blast

## Supplementary Material

FIGURE S1 **The cDNA and putative amino acid sequences of pepper *CaNAC2***.

FIGURE S2 **Amino acid sequences alignment of pepper CaNAC2 with others.** The five conserved motifs (A to E) are shown by thin underlines. The rich Ser regions in the C-terminal part are shown by thick underlines. The nuclear localization signal is lined with double arrows. The genes included are ANAC050 (XP_002882675.1), ANAC051/52 (XP_002882676.1), ANAC053 (NP_566376.1), ANAC078 (At5g04410), and NAC2 (AAF09254.1) from *Arabidopsis*, NH4 (AAM34767.1) from *Petunia*.
