# Supplementary figures and images for: Characterization and expression profile of CaNAC2 pepper gene
Source: Front Plant Sci. 2015 Sep 17;6:755. doi: 10.3389/fpls.2015.00755 (PMC4585251; doi:10.3389/fpls.2015.00755)

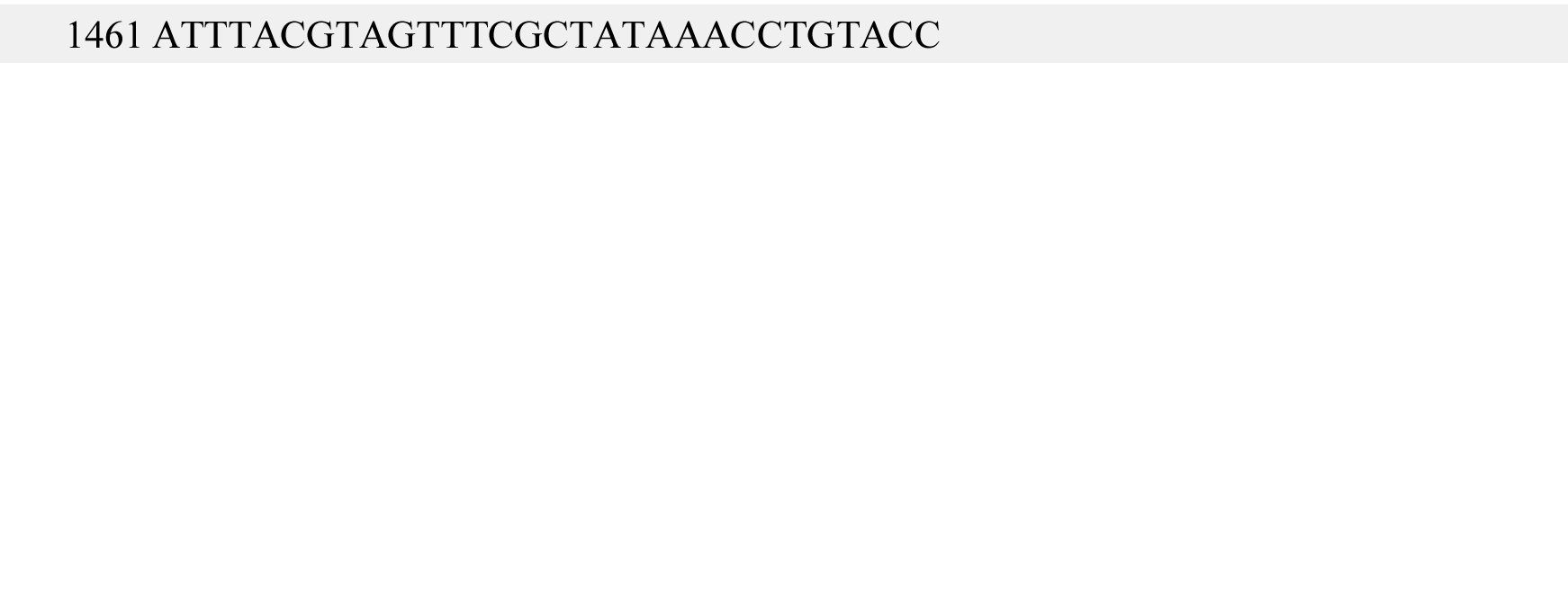

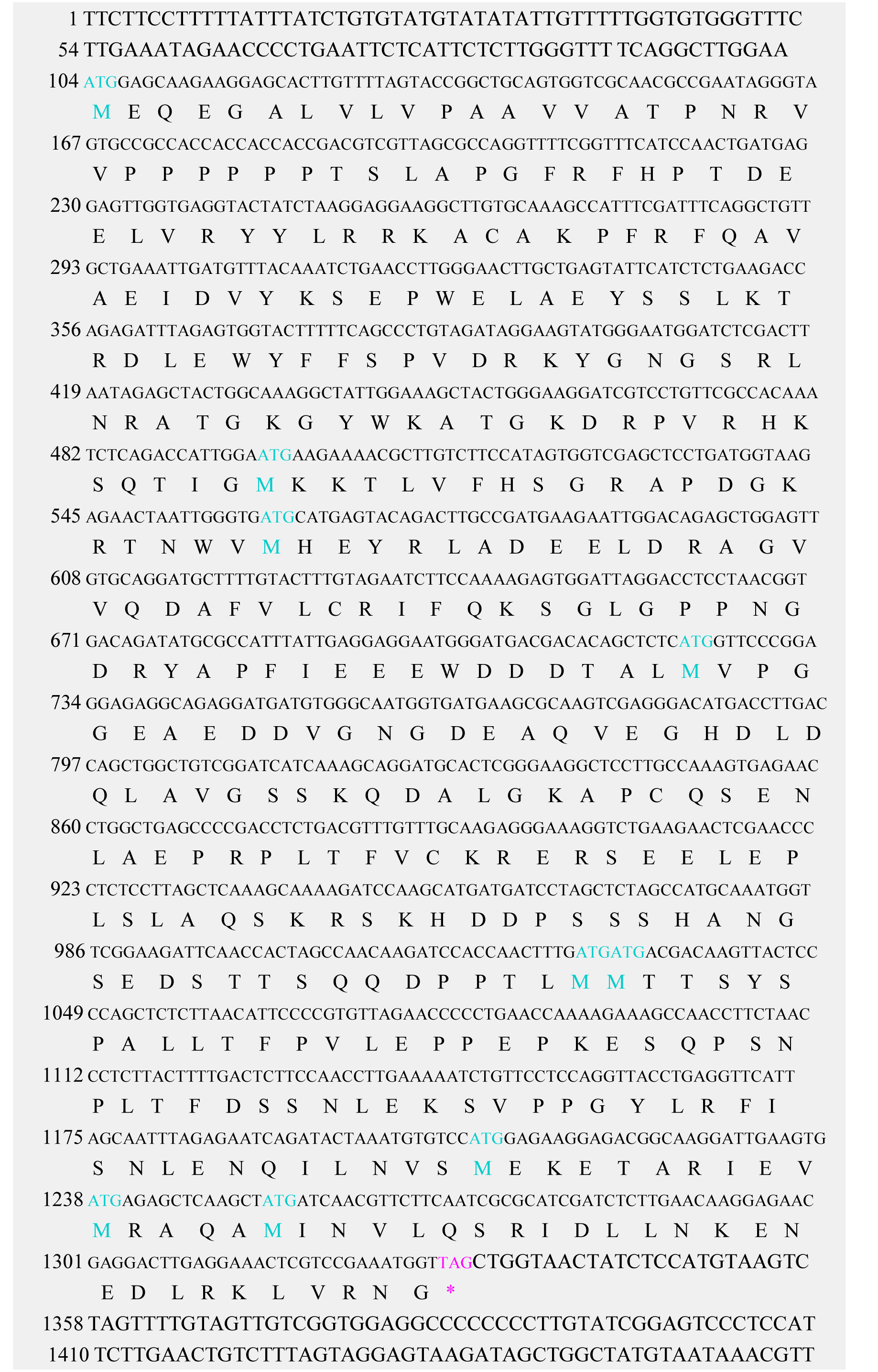

Supplement: FIGURE S1 — The cDNA and putative amino acid sequences of pepper CaNAC2. [file Data_Sheet_1.DOC]

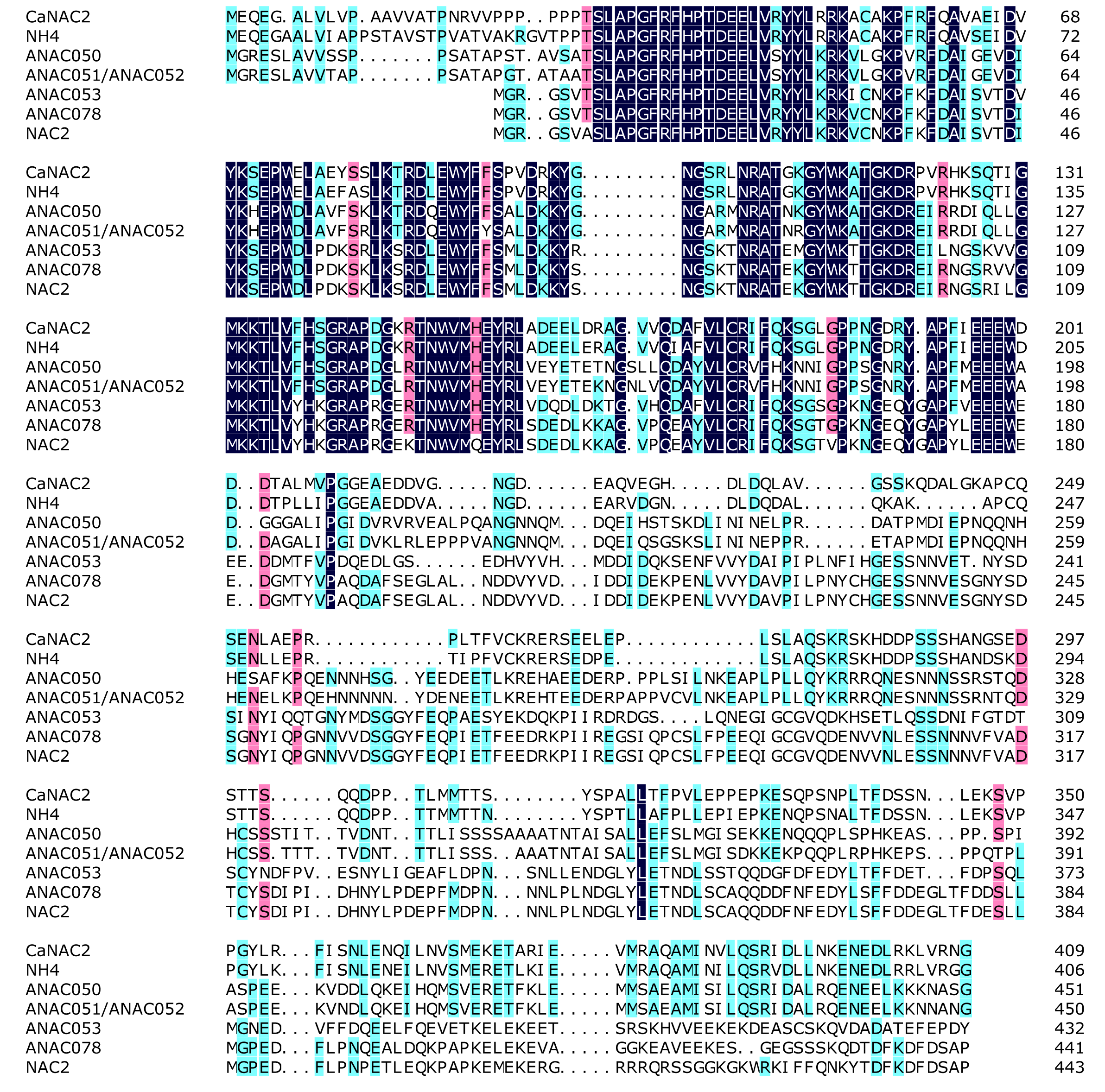

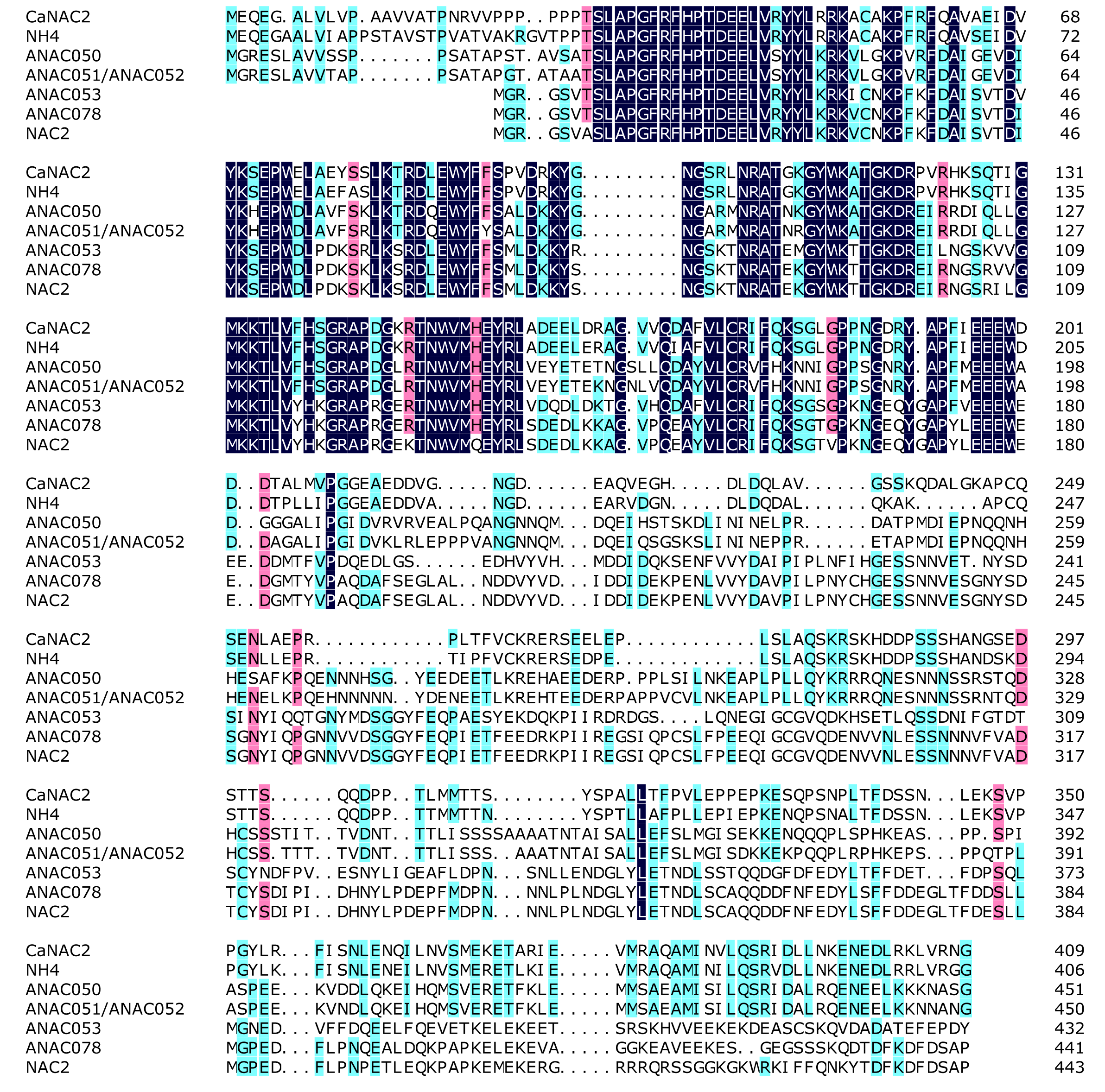

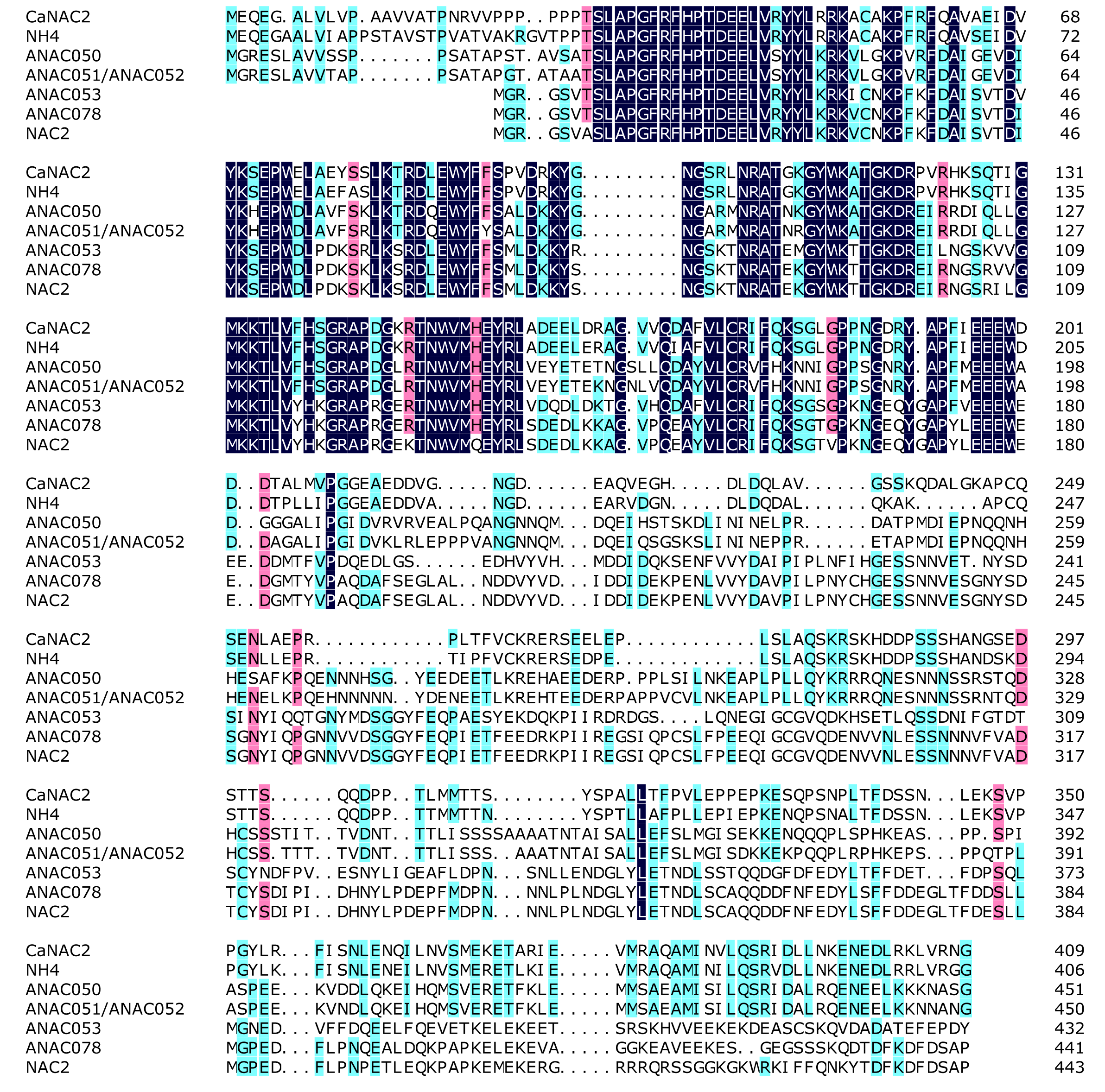

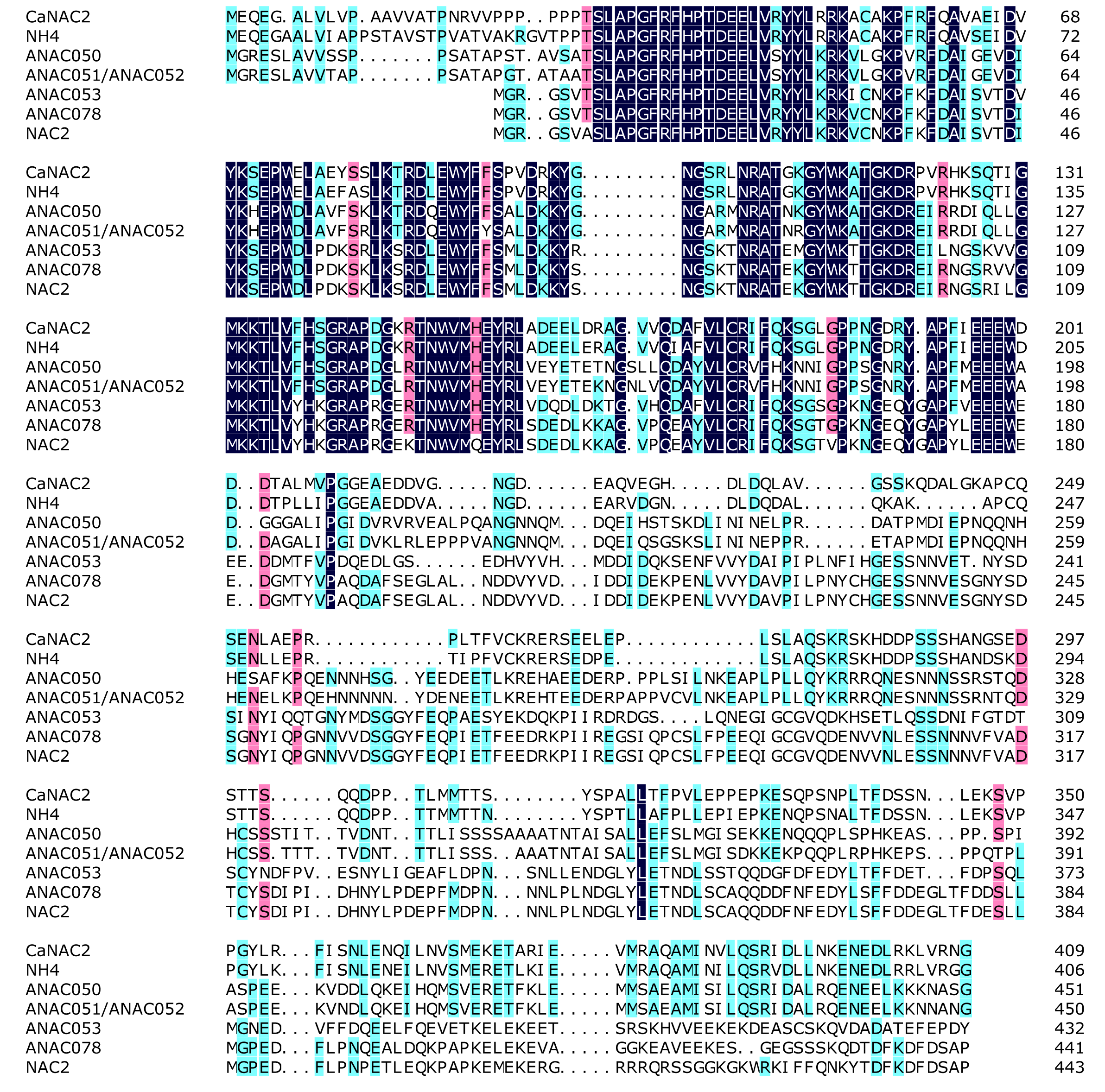

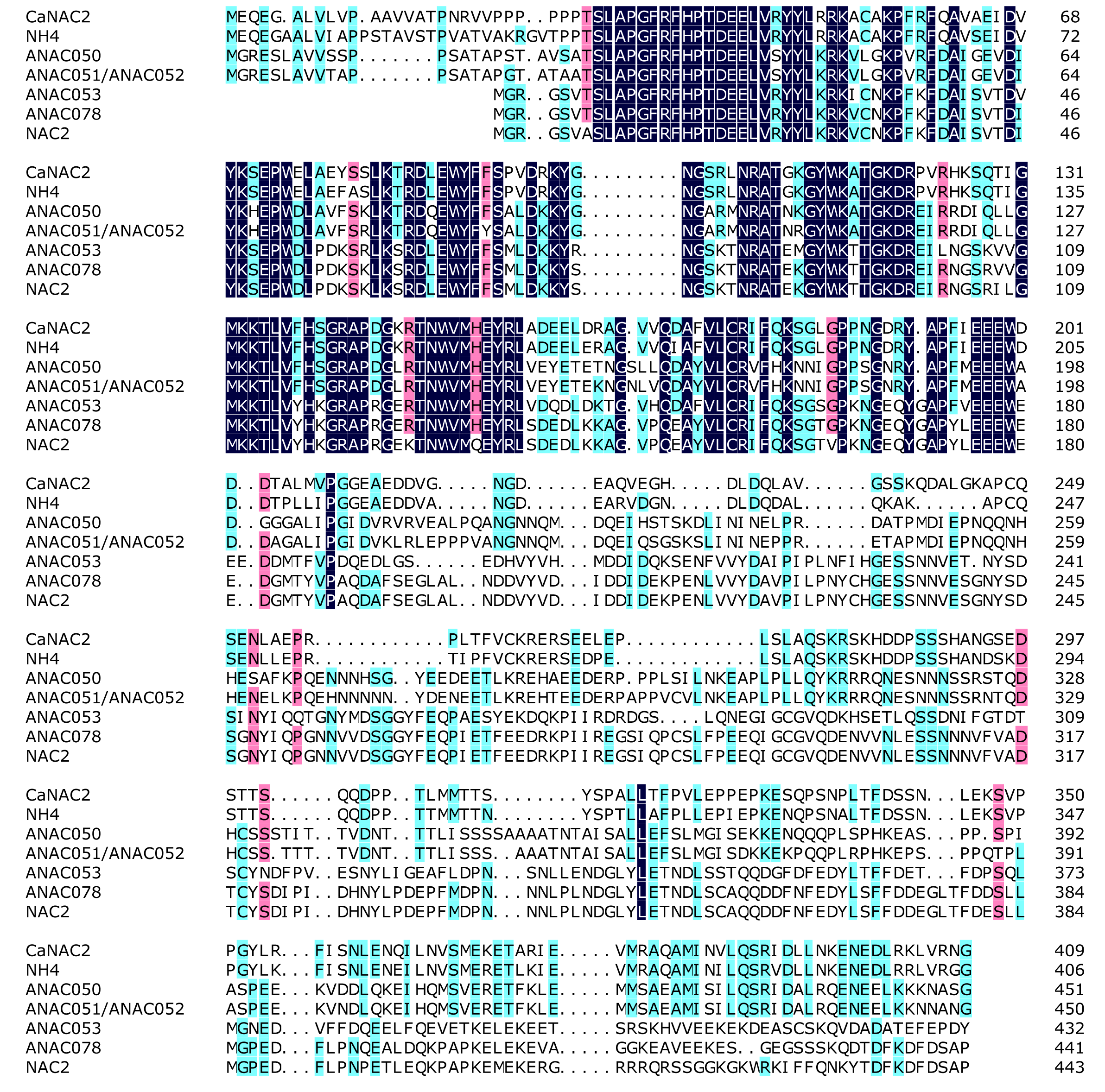


**B**


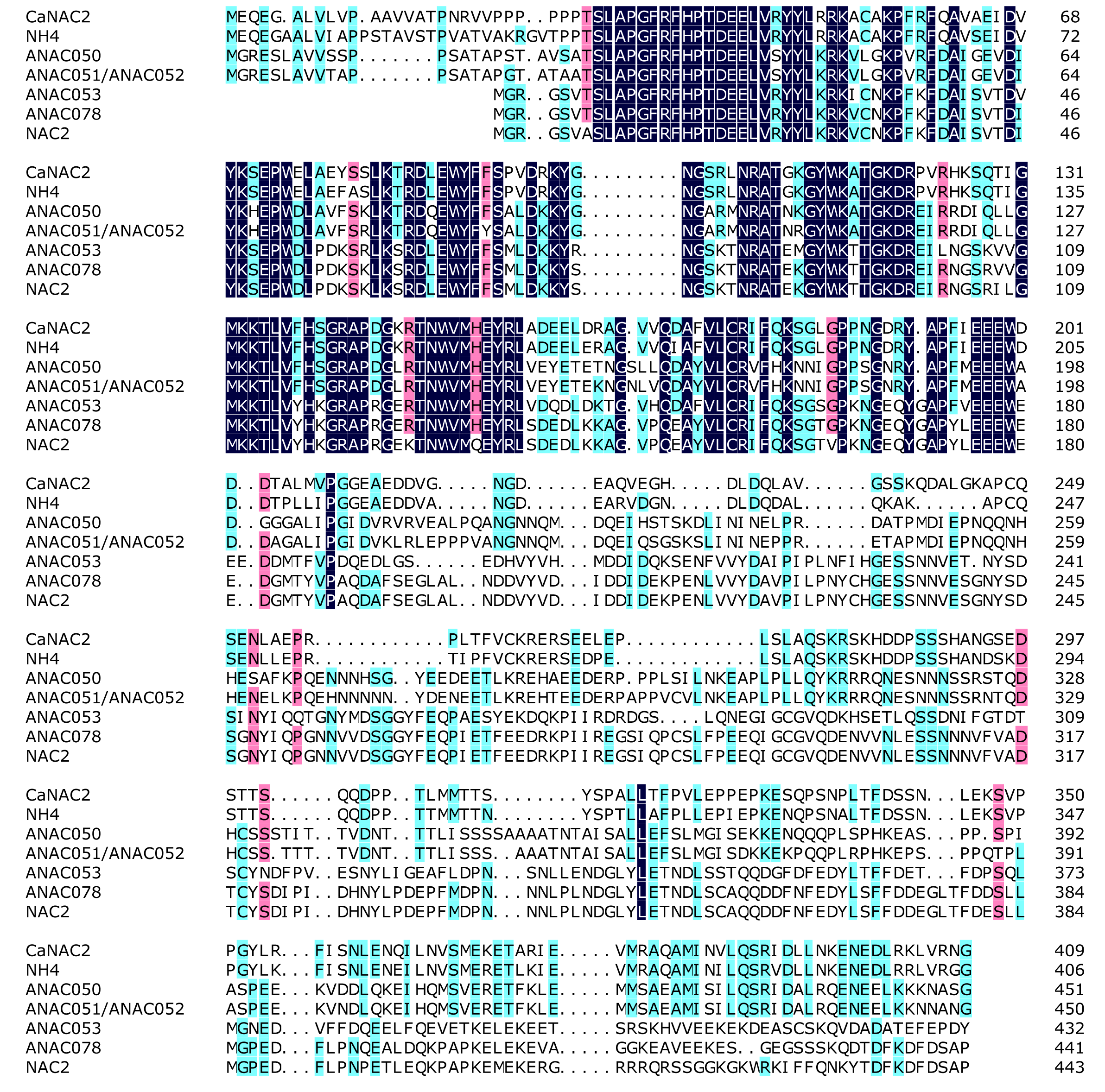


**D**


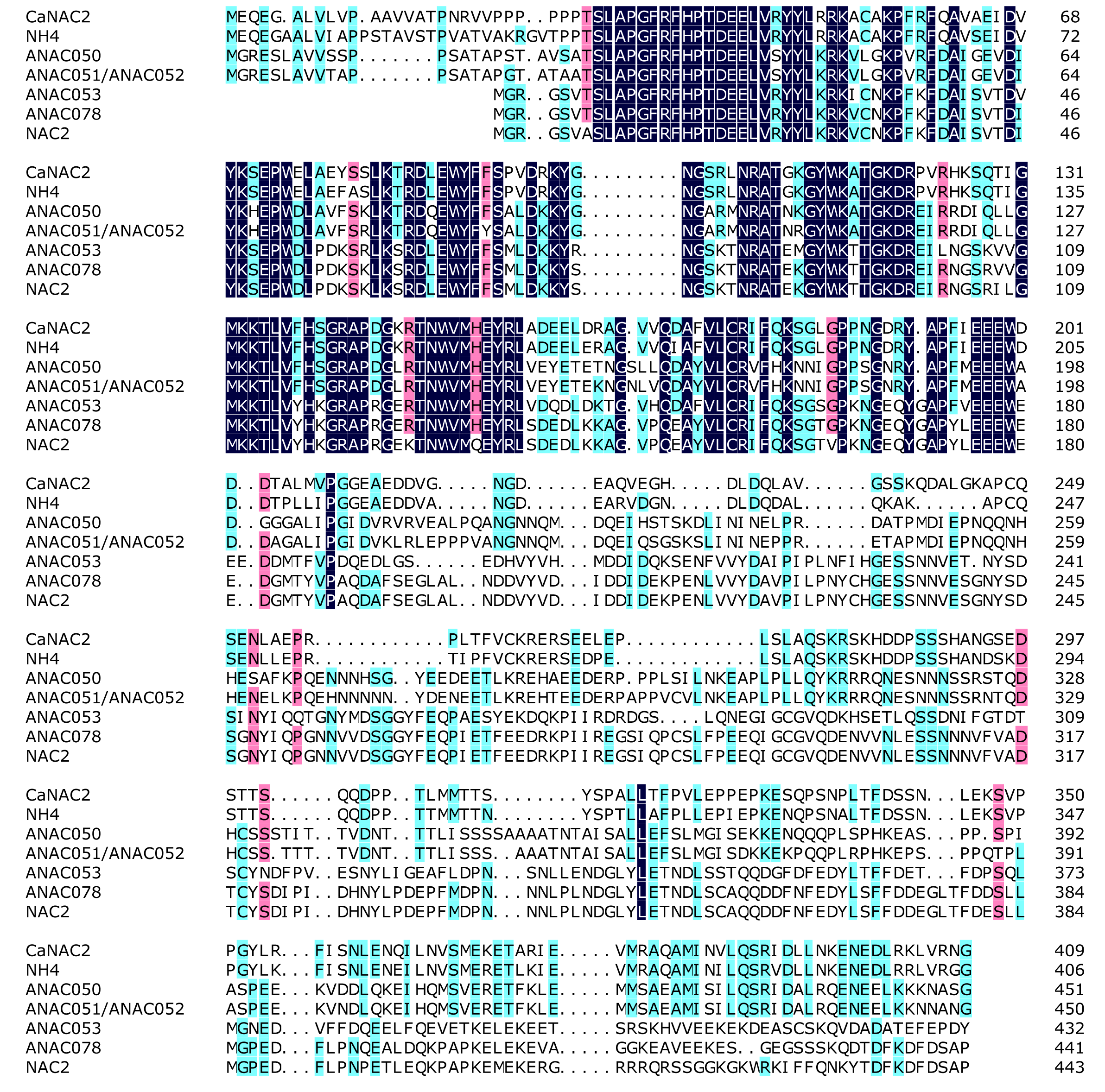


**A**

**C**

**E**

Supplement: FIGURE S2 — Amino acid sequences alignment of pepper CaNAC2 with others. The five conserved motifs (A to E) are shown by thin underlines. The rich Ser regions in the C-terminal part are shown by thick underlines. The nuclear localization signal is lined with double arrows. The genes included are ANAC050 (XP_002882675.1), ANAC051/52 (XP_002882676.1), ANAC053 (NP_566376.1), ANAC078 (At5g04410), and NAC2 (AAF09254.1) from Arabidopsis, NH4 (AAM34767.1) from Petunia. [file Data_Sheet_2.DOC]
